# Patient Perceptions of Vascular Access and Quality of Life in Maintenance Hemodialysis: A Multicenter Study on Patient-Centered Outcomes

**DOI:** 10.3390/healthcare14050697

**Published:** 2026-03-09

**Authors:** Eirini Eftychia Kokkinidi, Angeliki Chandrinou, Konstantinos Exarchos, Alexios Alexopoulos, Evangelos Fradelos, Aikaterini Toska, Maria Saridi, Maria Malliarou, Pavlos Sarafis

**Affiliations:** 1 Dialysis Unit, Athens Naval Hospital, 11521 Athens, Greece; ekokkinidi19@gmail.com; 2 Department of Health Care Management, Hellenic Open University, 26335 Patras, Greece; 3 Department of Nursing, University of Thessaly, 41500 Larissa, Greece; achandrinou@uth.gr (A.C.); efradelos@uth.gr (E.F.); ktoska@uth.gr (A.T.); malliarou@uth.gr (M.M.); 4 Anesthesiology Department, 251 General Airforce Hospital, 11525 Athens, Greece; 5 First Department of Paediatrics, School of Medicine, National and Kapodistrian University of Athens, “Agia Sofia” Children’s Hospital, 11527 Athens, Greece; atosmedicals@yahoo.co.uk

**Keywords:** chronic kidney disease, hemodialysis, vascular access, quality of life

## Abstract

**Highlights:**

**What are the main findings?**

- Vascular access type was associated with meaningful differences in patient-reported quality of life among maintenance hemodialysis patients, with arteriovenous fistula use linked to better scores in several SF-36 domains than permanent catheter use.
- Patient satisfaction with vascular access and selected sociodemographic/clinical factors (e.g., age, education, marital status, BMI, and dialysis duration) independently contributed to variability across physical and psychosocial quality-of-life dimensions.

**What are the implications of the main findings?**

- Vascular access planning should integrate clinical suitability with patient-centered outcomes, prioritizing fistula creation when feasible and monitoring access satisfaction as part of routine follow-up.
- Targeted supportive interventions may be warranted for patients at higher risk of poorer quality of life (e.g., those using catheters or reporting low access satisfaction), to improve symptom burden, engagement, and overall well-being.

**Abstract:**

**Background:** Vascular access is a core component of hemodialysis and may influence both clinical outcomes and patient-reported quality of life. This study examined the association between vascular access type and quality of life among patients receiving maintenance hemodialysis in multiple nephrology centers. **Methods:** We conducted a multicenter, cross-sectional observational study of 152 adults with end-stage kidney disease undergoing hemodialysis in public and private dialysis units in the Attica region, Greece (January–May 2022). Data were collected using a demographic/clinical questionnaire, the 36-Item Short Form Health Survey (SF-36), the Dialysis Patient Satisfaction Questionnaire (SDIALOR), and the Missoula VITAS Quality of Life Index (MVQOLI). Multivariable linear regression models were fitted for SF-36 and MVQOLI domain scores. **Results:** Most participants reported being very (40.8%) or quite (53.3%) satisfied with their current vascular access, and 69.5% considered an arteriovenous fistula (AVF) the most appropriate option. SF-36 scores were generally lower than those reported for the general population, except for the mental health domain. Compared with AVF, permanent catheter use was associated with lower SF-36 physical functioning scores, and graft use was associated with lower vitality scores. Lower vascular access satisfaction was consistently associated with lower HRQoL: compared with being “very” satisfied, being “quite” satisfied was associated with lower general health, vitality, social functioning, mental health, and lower PCS/MCS scores, while being “a little/not at all” satisfied was associated with lower general health and worse bodily pain scores. On MVQOLI, living alone and lower access satisfaction were associated with lower interpersonal relationships, transcendence/spirituality, and overall quality-of-life scores, while obesity was associated with lower function scores. **Conclusions:** Vascular access type, particularly AVF versus catheter, is associated with meaningful differences in quality of life among hemodialysis patients. Patient satisfaction with access and sociodemographic characteristics should be considered in patient-centered access planning and follow-up.

## 1. Introduction

### 1.1. Chronic Kidney Disease

Chronic kidney disease (CKD) is characterized by a progressive and often irreversible decline in kidney function arising from a range of pathological conditions. It is commonly defined by a reduction in glomerular filtration rate (GFR) to <60 mL/min/1.73 m^2^ and/or evidence of kidney damage persisting for more than three months, reflecting a substantial loss of functional nephrons [1,2]. CKD may remain asymptomatic for prolonged periods; however, typical manifestations include fatigue, impaired concentration, pruritus, peripheral or facial edema, anorexia that may be accompanied by nausea and vomiting, and sleep disturbances. Clinically, CKD is associated with electrolyte and fluid imbalances and with hematological, cardiovascular, bone–mineral, metabolic, neurological, and gastrointestinal complications [3].

### 1.2. Vascular Access

Initiation and continuation of maintenance hemodialysis require reliable vascular access to enable repeated extracorporeal circulation at adequate blood flow rates [4]. The main access options include arteriovenous fistulas (AVFs), arteriovenous grafts (AVGs), and central venous catheters (CVCs) [4]. In general, AVFs are preferred when feasible because they are associated with better long-term patency and fewer access-related complications, whereas AVGs may be used when native vessels are unsuitable [4,5]. CVCs are typically reserved for urgent dialysis initiation or as a bridge to permanent access; however, they are linked to higher risks of infection and thrombosis and may contribute to access-related complications [4,6]. Accordingly, an “ideal” vascular access should be safe, durable, and functional, supporting effective dialysis while aligning with patient preferences and quality-of-life priorities [5,7].

Contemporary guidance has shifted from a rigid “fistula-first” paradigm toward an individualized, patient-centered approach aligned with the patient’s end-stage kidney disease (ESKD) “life-plan”, emphasizing the right access for the right patient at the right time. In practice, this approach integrates expected dialysis trajectory, comorbidity burden and survival, vascular anatomy, and—critically—patient goals and preferences within a shared decision-making process [4,8]. As demonstrated by qualitative evidence, patients frequently prioritize dimensions that are not adequately addressed by conventional clinical endpoints. These include body image and appearance, limitations in bathing and water exposure, cannulation-related pain and anxiety, and confidence in access reliability. Proactively addressing these factors may enhance engagement and sustained satisfaction with vascular access care [9].

### 1.3. Quality of Life in Hemodialysis Patients

Quality of life (QoL) among patients undergoing maintenance hemodialysis is shaped by a complex interplay of physical, psychological, social, and economic determinants. ESKD and long-term dialysis treatment impose substantial constraints on everyday life, often limiting functionality, social participation, and personal autonomy, and thereby negatively affecting perceived QoL. In this context, QoL is not solely a reflection of clinical status, but also of how patients adapt to the ongoing demands and disruptions associated with chronic therapy. In addition, psychosocial resources such as spirituality and meaning-making have been proposed as factors that may influence perceived QoL among patients with chronic kidney disease [1].

Demographic characteristics appear to influence psychosocial adjustment and patients’ subjective experience of the disease. For example, sex and age have been linked to differences in coping and emotional responses: women tend to report a higher burden of anxiety and depressive symptoms than men, which may be partly related to the expectations and pressures of social roles, whereas older individuals often exhibit greater acceptance of the chronic nature of illness and report higher levels of adaptation and satisfaction [10].

Socioeconomic conditions also play a central role. Educational attainment, occupational status, and income have been associated with treatment adherence, health outcomes, and QoL in patients with end-stage chronic kidney disease, highlighting how social resources can facilitate or hinder effective long-term self-management [11]. The loss of employment—or the inability to maintain professional activity due to the limitations imposed by dialysis schedules, fatigue, and functional restrictions—may further burden psychological well-being and social functioning. Conversely, patients who remain professionally active have been reported to demonstrate better adherence, higher self-esteem, and improved QoL [12].

Beyond individual characteristics, the broader family and social environment is particularly important. Stable interpersonal relationships and marital status have been associated with better adjustment to chronic illness and enhanced psychosocial well-being, potentially through emotional support and practical assistance. Moreover, physical symptoms and clinical stressors—such as fatigue, reduced physical activity, treatment-related complications, and recurrent hospitalizations—can directly impair daily functioning and contribute to poorer QoL [13].

Finally, psychosocial problems, including depression, anxiety, and sexual dysfunction, have been identified as critical contributors to diminished QoL among hemodialysis patients. These factors may influence not only perceived well-being, but also adherence to treatment and the quality of communication and collaboration between patients and healthcare professionals [9,14].

In addition to overall health-related quality of life (HRQoL), a growing body of work emphasizes vascular access–specific patient-reported outcomes (PROs) as an essential complement to patency, adequacy, and complication rates. Access-related symptoms and burdens, including bruising and bleeding, fear of infection, restrictions in hygiene and water exposure, discomfort with needling, and impacts on social functioning, may substantially shape daily life and perceived well-being [11,15,16]. Instruments developed or validated in recent years, such as the Hemodialysis Access-Related Quality of Life (HARQ) instrument and the Vascular Access–Specific Quality of Life measure (VASQoL), enable a granular assessment of these patient-centered impacts [17,18].

Recent studies continue to report differences in HRQoL profiles across access types, often favoring arteriovenous fistulas over catheters on selected domains while also describing nuanced trade-offs (e.g., fistula-related discomfort versus catheter-related social restrictions) [11,16]. Nevertheless, findings vary by setting, patient characteristics, and outcome measures, and evidence from Southern European populations remains limited. Therefore, the present multicenter study in Attica, Greece, examined the association between vascular access type and patient-reported QoL using both a generic HRQoL measure (SF-36) and a broader quality-of-life tool (MVQOLI), while accounting for access satisfaction and key sociodemographic and clinical factors.

Despite growing interest in access-related patient-reported outcomes, prior studies have often focused on a single HRQoL instrument, have not simultaneously incorporated broader existential/psychosocial dimensions of quality of life, or have been conducted in single-center settings. The present study extends the literature by evaluating vascular access type alongside vascular access satisfaction and sociodemographic/clinical factors using a combination of a generic HRQoL measure (SF-36) and a multidimensional quality-of-life instrument (MVQOLI) in a multicenter cohort from Southern Europe (Attica, Greece), including both public and private dialysis units. This design enables a more comprehensive characterization of how access type and patients’ subjective experience of their access relate to both physical and psychosocial quality-of-life domains.

## 2. Materials and Methods

### 2.1. Aim

The primary objective of this cross-sectional, non-interventional observational study was to examine the association between vascular access type (arteriovenous fistula, arteriovenous graft, or permanent catheter) and patient-reported QoL among adults receiving maintenance hemodialysis. Secondary objectives were to assess whether vascular access satisfaction and selected demographic and clinical characteristics (e.g., age, sex, education, marital status, body mass index, dialysis duration, and laboratory parameters) were independently associated with quality-of-life outcomes. We hypothesized that arteriovenous fistula use and higher access satisfaction would be associated with more favorable quality-of-life scores.

### 2.2. Research Approval

Administrative permission to conduct the study was obtained from the management of the participating dialysis units. The study was conducted in accordance with the Declaration of Helsinki and relevant national data-protection regulations. All participants were informed about the study and provided written informed consent. Data were collected anonymously, and results are reported only in aggregate form.

### 2.3. Study Design

This multicenter, cross-sectional, non-interventional observational study collected data at a single time point from adults receiving maintenance hemodialysis. No changes were made to routine clinical care or vascular access management. The study was conducted in the Attica region of Greece, and data collection took place between January 2022 and May 2022.

### 2.4. Study Population

Participants were recruited from one public hemodialysis unit (the Dialysis Unit of the Naval Hospital of Athens) and four private dialysis centers (Mesogeios Chaidariou, Mesogeios Paleo Faliro, Nefros Nea Ionia, and Nephromedica Metamorfosis), all located in the Attica region of Greece. Eligible participants were adults (≥18 years) with ESKD receiving maintenance hemodialysis. Exclusion criteria were (a) hemodialysis for acute kidney failure and (b) patients in the initiation phase of hemodialysis treatment. Participation was voluntary and anonymous. Consecutive eligible patients were approached during routine dialysis sessions.

Patients were eligible regardless of prior vascular access history; therefore, participants with previous access creations, failures, or conversions (e.g., prior fistulas/grafts or prior catheter use) were included. Patient-reported outcomes and satisfaction were assessed in relation to the vascular access in use at the time of the survey.

### 2.5. Data Collection

Data were collected between January 2022 and May 2022 using a pack of self-administered questionnaires. Eligible participants were approached during their attendance at the participating dialysis units, and questionnaires were distributed by the study team (in some cases with the assistance of dialysis staff). Participants could complete the questionnaires either on-site or off-site, according to their preference. Completed questionnaires were returned either immediately after completion or within five days of distribution. To preserve anonymity, no direct personal identifiers were collected, and responses were analyzed in aggregate form. Questionnaires were checked for completeness upon return, and missing responses were handled as described in the Statistical Analysis Section.

### 2.6. Data Collection Tool

The data collection instrument consisted of three sections. The first section captured demographic, social, and clinical characteristics (e.g., age, sex, marital status, educational level, dialysis duration, laboratory parameters, and vascular access type).

Health-related quality of life (HRQoL) was assessed using the 36-Item Short Form Health Survey (SF-36), a widely used generic instrument comprising eight domains: physical functioning, role limitations due to physical health, bodily pain, general health, vitality, social functioning, role limitations due to emotional problems, and mental health. Domain scores are transformed to a 0–100 scale, with higher scores indicating better perceived health [19].

Patient satisfaction with dialysis care was assessed using the SDIALOR (Dialysis Patient Satisfaction Questionnaire), a multidimensional instrument designed to evaluate patient satisfaction across key aspects of dialysis care; scores can be standardized on a 0–100 scale, with higher scores reflecting greater satisfaction [20].

Overall QoL was additionally assessed using the MVQOLI, a patient-centered instrument that evaluates five core dimensions (symptoms, function, interpersonal relationships, well-being, and transcendence/spirituality) while accounting for the importance attributed to each dimension by the respondent. Higher scores indicate better perceived QoL, and some dimension scores may take negative values [21]. The MVQOLI was selected to complement generic HRQoL assessment because it captures broader, patient-centered dimensions of quality of life, including interpersonal relationships, well-being, and transcendence/spirituality, that may be particularly relevant in long-term, life-sustaining therapies such as maintenance hemodialysis. This approach enabled us to assess aspects of lived experience that are not fully represented by symptom- and function-focused instruments alone.

### 2.7. Statistical Analysis

Quantitative variables are presented as mean ± standard deviation (SD), and qualitative variables as absolute and relative frequencies (n, %). The normality of continuous variables was assessed using the Kolmogorov–Smirnov test. To examine factors associated with PROs, multiple linear regression analyses were performed using SF-36 domain scores (and summary component scores, where applicable) and MVQOLI dimension scores (and the overall MVQOLI score) as dependent variables. Demographic and clinical characteristics were entered as independent variables. Comorbidity indices (e.g., Charlson Comorbidity Index) and detailed vascular anatomy measures were not available for all participants and were therefore not included in the regression models. Categorical predictors were included using indicator (dummy) coding with clearly defined reference categories. Adjusted regression coefficients (β) with corresponding 95 confidence intervals (95% CI) are reported. Multicollinearity was diagnosed via VIF and tolerance indexes. Linearity, homoscedasticity and normality of error distribution were checked graphically, via scatterplots and Q-Q plots. Independence of errors was checked via the Durbin–Watson test. No imputation of the missing data was performed. All tests were two-tailed, and statistical significance was set at *p* < 0.05. Analyses were conducted using SPSS (version 24.0). Model assumptions were evaluated using standard diagnostic procedures. Generative AI (OpenAI; GPT-5.2; accessed in January 2026) was used to assist in drafting and language refinement of selected passages and to support the preparation of the graphical abstract; all content was reviewed and edited by the authors.

## 3. Results

The sample consisted of 152 participants whose demographic and clinical characteristics are presented in Table S1. Of the participants, 65.8% were men and 71.1% were over 56 years of age. Additionally, 82.9% were married or living with a partner, and 55.2% were graduates of middle school or high school. 40.8% were overweight, while regarding hemodialysis history, 42.1% had been undergoing treatment for more than five years, while the mean values of the most recent laboratory measurements for urea and creatinine were 134.6 units (SD = 29.3) and 8.3 (SD = 2.6), respectively. Furthermore, 59.2% of the participants had an arteriovenous fistula and 27% had a permanent dialysis catheter. Concerning satisfaction with their current vascular access, 53.3% reported being quite satisfied, and 69.5% identified the arteriovenous fistula as the most appropriate vascular access for themselves.

QoL was assessed using the SF-36 and MVQOLI scales, the descriptive results of which are presented in Table S2. Among the SF-36 dimensions, the highest mean values were observed in Bodily Pain (67.9, SD = 24.1), Mental Health (66.2, SD = 17.5), and Social Functioning (66.0, SD = 23.1). Additionally, the mean score for the Physical Health Summary Scale was 40.9 (SD = 9.4), while the mean score for the Mental Health Summary Scale was 46.4 (SD = 9.2). The MVQOLI scale showed a mean score of 6.7 (SD = 7.6) in the Symptoms dimension and 7.3 (SD = 8.2) in the Functionality dimension. The Interpersonal Relationships dimension demonstrated a higher mean score of 15.3 (SD = 10.0), while the Well-being dimension had a mean score of –6.6 (SD = 13.9). The Spirituality dimension recorded a mean value of 9.8 (SD = 12.1). Finally, the overall MVQOLI quality-of-life score had a mean value of 18.2 (SD = 3.0).

To identify the factors associated with QoL, multiple linear regression analyses were performed, using each quality-of-life dimension as a dependent variable and the demographic and clinical characteristics of participants as independent variables (Table S3). Age and type of vascular access were independently associated with the “Physical Functioning” dimension, as participants older than 56 years had a lower score than those aged 18 to 55 years, while those with a permanent catheter had a lower score than individuals with an arteriovenous fistula. Educational level was the only factor associated with “Role Physical”, with having a secondary-education being associated with higher score than having no education or only primary schooling. Years on hemodialysis and satisfaction with the current vascular access were related to “Bodily Pain”; being on hemodialysis for six months to five years was associated with higher score—indicating less pain—than being on treatment for less than six months, whereas being not at all or only slightly satisfied with their access was significantly associated with lower score than being very satisfied. Satisfaction with the vascular access was also linked to “General Health”, with lower scores observed among participants who were not at all, slightly, or moderately satisfied. Both the type of vascular access and satisfaction with it were independently associated with “Vitality” and “Social Role”. Having a graft was associated with lower vitality than having a fistula and being moderately satisfied was associated with lower score than being very satisfied. Similarly, having a permanent catheter was associated with lower score in social role, and being moderately satisfied with lower score than being very satisfied. Educational level alone was associated with “Role Emotional”, as having secondary-education being associated with higher score than having no or only primary education. Educational level and satisfaction with vascular access were also associated with “Mental Health”, with having university degree being associated with higher score than having no education or only primary education, while being moderately satisfied was associated with lower score than being very satisfied. Satisfaction with the vascular access further influenced the “Mental Health Summary Scale”, where being moderately satisfied was associated with lower score than being very satisfied. Finally, years on hemodialysis and satisfaction with vascular access were independently related to the “Physical Health Summary Scale”. Being on hemodialysis for six months to five years was associated with higher score—indicating better physical health—than being on treatment for less than six months, whereas being moderately satisfied was significantly associated with lower score than being very satisfied.

From the corresponding analyses of the MVQoL dimensions and the overall scale, sex and educational level were independently associated with the “Symptoms” dimension. Femininity was associated with lower score, while having secondary or higher education was associated with higher score than having no or only primary education. Body Mass Index (BMI) was the only independent predictor of the “Functionality” dimension, with obesity being significantly associated with lower scores—indicating poorer functional ability—than having a normal BMI. Family status and satisfaction with the current vascular access were associated with “Interpersonal Relationships”. Living alone and being not at all or only slightly satisfied with the vascular access were associated with lower scores. No factors were independently associated with “Well-being”. “Spirituality” was associated with family status, satisfaction with vascular access, and participants’ preferences regarding the most suitable access type. Living alone was associated with lower score, while being not at all, slightly, or moderately satisfied was associated with lower score than being very satisfied. Considering a permanent catheter as the most appropriate access option was associated with lower score than considering an arteriovenous fistula as appropriate access option. Finally, overall QoL was independently associated with family status, education, and satisfaction with vascular access. Living alone was associated with lower score; university graduates with higher score, and being not at all, slightly, or moderately satisfied with lower scores compared with being very satisfied (Table S4).

No multicollinearity issues were present (VIF ranged from 1.11 to 4.97 and tolerance from 0.20 to 0.90). All Durbin–Watson d statistics were close to 2, indicating that there is independence among the errors. Linearity and homoscedasticity were checked graphically with the appropriate scatterplots and normality of error distribution with Q-Q plots. Overall, no significant violations of the linear regression assumptions were found.

## 4. Discussion

Chronic kidney disease (CKD) is a growing global public health challenge, associated with substantial morbidity and mortality [3]. For patients receiving maintenance hemodialysis, the treatment burden extends beyond clinical outcomes, affecting physical functioning, psychological well-being, social participation, and occupational roles, ultimately compromising health-related quality of life (HRQoL) [10,22]. Within this framework, vascular access is not only a technical prerequisite for dialysis delivery but also a potentially modifiable factor that may shape patients’ daily experiences and PROs.

In this multicenter cohort, vascular access type was associated with meaningful differences in patient-reported QoL. Overall satisfaction with vascular access was high, and most participants identified the arteriovenous fistula (AVF) as the most appropriate option. These findings are consistent with prior evidence suggesting that AVFs are generally linked to fewer access-related complications and higher patient satisfaction compared with grafts and central venous catheters, and with studies reporting more favorable quality-of-life outcomes among patients dialyzing with an AVF than among those using catheter-based access [5,7].

Patients dialyzing with an arteriovenous fistula (AVF) reported higher HRQoL in several SF-36 domains compared with those using permanent catheters, including physical functioning, general health, and social functioning, as well as a higher physical component summary score. Compared with arteriovenous grafts, AVF use was also associated with higher vitality and social functioning. These patterns may reflect differences in access-related burden: catheter-based access is more frequently associated with infection and thrombosis risk, care restrictions, and heightened concerns about access safety, which can limit physical activity and social engagement. In contrast, a well-functioning AVF may support more stable dialysis delivery with fewer disruptions to daily routines, translating into better perceived functional capacity and participation.

Although AVF use was associated with more favorable HRQoL in several domains, patient preferences may not always align with these average patterns. Some patients may prefer catheter-based access despite its association with lower physical functioning and greater access-related burden, particularly when prioritizing short-term convenience, avoidance of cannulation, perceived comfort, or reduced concerns about appearance and repeated needling. Prior negative experiences with fistulas or grafts (e.g., failed maturation, painful cannulation, or repeated interventions), limited vascular anatomy, and the cumulative burden of long-term treatment may further shape preferences toward catheters. These trade-offs underscore that access choice is not solely a clinical decision but also a values-based decision; therefore, shared decision making should explicitly explore patient priorities, anticipated lifestyle impact, and tolerance for access-related risks and burdens. In our cohort, the high overall satisfaction and the fact that not all participants identified AVF as the preferred option further support the need for individualized discussions that reconcile clinical evidence with patient goals.

Beyond vascular access type, several sociodemographic and clinical characteristics were independently associated with quality-of-life outcomes in our sample. Older age and lower educational attainment were related to poorer physical and emotional role functioning, suggesting reduced capacity to maintain usual activities and psychosocial roles. Longer dialysis duration and lower satisfaction with vascular access were associated with greater bodily pain and poorer general health perceptions, indicating that both treatment vintage and access-related experience may shape everyday symptom burden and perceived health status. Importantly, family status and vascular access satisfaction were also linked to psychosocial domains of the MVQOLI, including interpersonal relationships, spirituality, and overall QoL, with participants living alone or expressing lower access satisfaction consistently reporting worse scores. The MVQOLI transcendence/spirituality domain warrants particular attention in a maintenance hemodialysis context, as it reflects meaning-making, existential well-being, and the extent to which individuals draw on values, beliefs, or a sense of purpose when living with a chronic, life-sustaining therapy. In our cohort, lower scores in this domain among participants living alone and those reporting lower vascular access satisfaction may indicate that reduced social support and greater access-related burden can undermine coping resources and perceived meaning in daily life. This interpretation is consistent with broader evidence suggesting that psychosocial resources, including spirituality and perceived purpose, may buffer distress and contribute to perceived quality of life in chronic kidney disease. At the same time, spirituality is culturally embedded and may vary substantially between individuals; therefore, future longitudinal studies should examine how transcendence/spirituality evolves over time and whether supportive interventions (e.g., psychosocial or spiritual care, peer support, or counseling) can improve this dimension, particularly following access complications or transitions. Overall, these findings support the multifactorial nature of HRQoL in hemodialysis and align with prior evidence highlighting the contribution of demographic, socioeconomic, and psychosocial factors to patient-reported well-being in ESKD [10].

Collectively, our findings suggest that QoL in maintenance hemodialysis is shaped by both clinical and experiential factors. Beyond access type, access satisfaction, treatment vintage, and sociodemographic characteristics contributed to variability across physical and psychosocial domains. These results highlight the relevance of patient-centered considerations in vascular access planning and follow-up, including clinical suitability, patient preferences, and patients’ reported access-related experience.

To contextualize our findings within contemporary evidence, we performed an updated targeted literature search of PubMed and Scopus (January 2019–December 2025) using combinations of terms related to hemodialysis, vascular access (fistula/graft/catheter), patient satisfaction/perspectives, and QoL/PROs. We prioritized systematic reviews/meta-analyses, multicenter observational studies, and validation studies of vascular access–specific patient-reported outcome measures (PROMs) [2,4].

Overall, our findings align with the patient-centered direction of recent guidance, which emphasizes individualized access planning guided by an ESKD “life-plan” and shared decision making rather than a one-size-fits-all approach [4]. In this framework, differences in patient-reported outcomes across access types should be interpreted alongside access-related burdens and patient priorities, which may vary substantially between individuals and settings [11,23].

Importantly, emerging work increasingly positions access satisfaction and access-specific PROs as key indicators of patient-centered vascular access success. Longitudinal evidence suggests that higher vascular access satisfaction is associated with better HRQoL trajectories over time, reinforcing the value of incorporating satisfaction and perceived access-related burden into routine follow-up and quality improvement [16]. Furthermore, recent systematic syntheses of patient perspectives indicate that satisfaction differs by access type across multiple domains (e.g., symptoms, social functioning, complications), supporting routine, structured assessment alongside traditional clinical endpoints [24]. Taken together, these data strengthen the interpretation of our results and suggest that validated access-specific measures could improve the granularity of patient-reported assessment in future studies and quality-improvement initiatives [17,18].

### 4.1. Recommendations for Practice

Based on the findings of this study and contemporary evidence, several practice-oriented considerations may be useful. Where clinically feasible, arteriovenous fistula creation should be prioritized and supported through timely referral and coordinated care, given its association with more favorable patient-reported quality-of-life outcomes compared with catheter-based access. Patient satisfaction with vascular access should also be routinely assessed as part of follow-up, as it may capture access-related burdens that are not fully reflected in clinical metrics and may help identify patients who would benefit from additional education, symptom management, or access troubleshooting. Vascular access planning should adopt a patient-centered approach that incorporates not only anatomical and clinical criteria but also patients’ preferences, lifestyle demands, and psychosocial context, including social support. Finally, multidisciplinary collaboration among nephrologists, vascular surgeons, and dialysis nurses is essential to optimize access selection and maintenance and to support patient education, with the goal of minimizing complications and improving functional status and overall well-being.

### 4.2. Limitations of the Study

This study should be interpreted considering several limitations. First, the cross-sectional design precludes causal inference regarding the relationship between vascular access type and patient-reported QoL. Second, participation was voluntary and recruitment occurred during routine dialysis sessions; therefore, selection bias cannot be excluded. Patients who felt more engaged with care, had stronger opinions about their vascular access, or were more comfortable completing questionnaires may have been more likely to participate. In addition, perceptions of the current access may have been shaped by previous vascular access experiences (e.g., prior access failures or complications), which were not fully quantified and may have influenced patient-reported outcomes. In addition, confounding by indication is possible, as patients dialyzing with permanent catheters may represent a more clinically complex subgroup (e.g., limited vascular anatomy, urgent dialysis initiation, higher comorbidity burden, or prior access failures) for whom fistula or graft creation was not feasible. Because comorbidity indices and detailed vascular anatomy variables were not consistently available, residual confounding cannot be excluded, and access-type associations should be interpreted cautiously. Third, although access-specific instruments have been developed (e.g., HARQ and VASQoL), we did not administer a vascular access–specific patient-reported outcome measure in this study. This may have reduced sensitivity to access-related symptoms and functional burdens (e.g., cannulation discomfort, restrictions in daily activities, and access-related anxiety) and limited the granularity of our patient-reported assessment. Instead, we assessed vascular access satisfaction using a brief, general measure to minimize respondent burden and maximize feasibility in a multicenter routine-care setting. While satisfaction provides a pragmatic summary of the patient experience, it does not capture the full spectrum of access-specific impacts. Fourth, although multiple public and private dialysis units were included, the sample size was modest, which may have limited statistical power for some comparisons. Fifth, data collection occurred during the COVID-19 pandemic, which may have affected participation and may have amplified the risk of selection bias. Pandemic-related restrictions (e.g., limited visitors, reduced social contact, and heightened uncertainty) may have disproportionately affected psychosocial domains of quality of life, including social functioning, interpersonal relationships, and emotional well-being. As a result, some psychosocial scores may reflect not only dialysis and access-related factors but also the broader contextual impact of the pandemic during the study period. Sixth, outcomes were based on self-reported questionnaires and are therefore susceptible to reporting bias. Due to the exploratory and cross-sectional design of the study and the multiple regression analyses performed across QoL domains, there is an increased risk of Type I error. The findings should therefore be interpreted cautiously and viewed as preliminary and hypothesis-generating. Replication in larger, confirmatory studies with correction for multiple testing is warranted. Finally, as participants were recruited from dialysis units within a single geographic region (Attica, Greece), the external validity of the findings may be limited, and caution is warranted when extrapolating results to different healthcare settings.

## 5. Conclusions

This multicenter study highlights that vascular access is not only a technical requirement for hemodialysis but also a patient-experienced factor linked to quality of life. Beyond access type, patients’ satisfaction with their access and their sociodemographic context appear to shape both physical and psychosocial dimensions of well-being. Future longitudinal research is needed to examine how quality of life evolves over time, particularly before and after vascular access transitions (e.g., catheter-to-fistula conversion, access failure, or repeated interventions), and to clarify whether patient-centered access strategies can produce sustained improvements in patient-reported outcomes.

## Data Availability

The aggregate data supporting the findings of this study are included in the article and/or the Supplementary Materials. The raw data are available from the corresponding author upon reasonable request. The raw data are not publicly available due to ethical and privacy considerations in accordance with the General Data Protection Regulation (GDPR), as they contain sensitive health-related information.

## Supplementary Materials

The following supporting information can be downloaded at https://www.mdpi.com/article/10.3390/healthcare14050697/s1, Supplemental Table S1: Demographic and clinical characteristics; Supplemental Table S2: Descriptive statistics of SF-36 and MVQoL dimensions; Supplemental Table S3: Results of multiple linear regression analysis with dependent variables of the dimensions of SF-36 scale; Supplemental Table S4: Results of multiple linear regression analysis with dependent variables of the dimensions of MVQoL scale.

## Abbreviations

The following abbreviations are used in this manuscript:

AVF	Arteriovenous fistula
AVG	Arteriovenous graft
BMI	Body mass index
CKD	Chronic kidney disease
CVC	Central venous catheter
ESKD	End-stage kidney disease
GFR	Glomerular filtration rate
HRQoL	Health-related quality of life
MVQOLI	Missoula–VITAS Quality of Life (Index)
QoL	Quality of life
PROs	Patient-reported outcomes
PROMs	Patient-reported outcome measures
SARS-CoV-2	Severe acute respiratory syndrome coronavirus 2
SD	Standard deviation
SDIALOR	Dialysis Patient Satisfaction Questionnaire
SE	Standard error
SF-36	36-Item Short Form Health Survey
SPSS	Statistical Package for the Social Sciences
