# Supplementary material for: Patient Perceptions of Vascular Access and Quality of Life in Maintenance Hemodialysis: A Multicenter Study on Patient-Centered Outcomes
Source: Healthcare (Basel). 2026 Mar 9;14(5):697. doi: 10.3390/healthcare14050697 (PMC12984611; doi:10.3390/healthcare14050697)
Supplement: Supplementary file 1 [file healthcare-14-00697-s001.zip › healthcare-4142450-supplementary.pdf]

Table S1. Demographic and clinical characteristics

		N	%
Gender	Male	100	65.8
	Female	52	34.2
BMI	Normal	57	37.5
	Overweight	62	40.8
	Obese	33	21.7
Age	18-55	44	28.9
	≥ 56	108	71.1
Marital status	Single	26	17.1
	In a relationship/married	126	82.9
Educational level	No education / Primary school graduate	34	22.4
	Middle school / High school graduate	84	55.2
	University degree / Master / PhD	34	22.4
How long have you been on hemodialysis?	≤ 6 months	10	6.6
	6-12 months	9	5.9
	1-2 years	22	14.5
	2-5 years	47	30.9
	≥ 5 years	64	42.1
Most recent urea lab values, Mean (SD)		134.6 (29.3)	
Most recent creatinine lab values, Mean (SD)		8.3 (2.6)	
What type of vascular access do you currently have?	Arteriovenous fistula	90	59.2
	Graft	14	9.2
	Temporary dialysis catheter	7	4.6
	Permanent dialysis catheter	41	27
Are you satisfied with your vascular access?	Very	62	40.8
	Quite	81	53.3
	A little	8	5.3
	Not at all	1	0.7
Which vascular access is considered appropriate for you?	Arteriovenous fistula	105	69.5
	Graft	16	10.6
	Temporary dialysis catheter	3	2
	Permanent dialysis catheter	27	17.9
In which hospital or center do you regularly receive your treatment?	Bioclinic	14	10.0
	Bionefros	7	5.0
	IASO Thessaly	1	0.7
	Mesogeios	62	44.3
	Nefroiatriki	33	23.6
	Nefros	7	5.0
	NNA	16	11.4

Table S2. Descriptive statistics of SF-36 and MVQoL dimensions

	Minimum	Maximum	Mean	SD
SF-36				
1. Physical functioning	0	100	57.9	25.1
2. Role functioning	0	100	54.1	43.3
3. Bodily pain	10	100	67.9	24.1
4. General health	5	207	51.4	22.6
5. Vitality	5	100	56.1	16.8
6. Social function	0	100	66.0	23.1
7. Role emotional	0	100	60.7	43.8
8. Mental health	16	100	66.2	17.5
Physical component score (PCS)	16.7	59.1	40.9	9.4
Mental component score (MCS)	18.1	66.4	46.4	9.2
MVQoL				
Symptoms	-16	30	6.7	7.6
Function	-15	25	7.3	8.2
Interpersonal relationships	-25	30	15.3	10.0
Emotional well-being	-30	30	-6.6	13.9
Transcendence	-30	30	9.8	12.1
Overall quality of life score	8.6	26	18.2	3.0

Table S3. Results of multiple linear regression analysis with dependent variables of the dimensions of SF-36 scale.

	Physical functioning		Role functioning		Bodily pain		. General health	
	β (95% CI)	P	β (95% CI)	P	β (95% CI)	P	β (95% CI)	P
Gender								
Male (reference)								
Female	-2.38 (-10.76 — 6)	0.575	-1.26 (-16.42 — 13.89)	0.869	-6.2 (-14.52 — 2.13)	0.143	-1.04 (-8.82 — 6.74)	0.794
BMI								
Normal (reference)								
Overweight	-3.46 (-12.57 — 5.66)	0.454	-4.33 (-20.82 — 12.16)	0.605	0.8 (-8.25 — 9.86)	0.861	5.35 (-3.12 — 13.82)	0.217
Obese	-6.68 (-17.19 — 3.83)	0.211	-5.74 (-24.76 — 13.27)	0.551	2.08 (-8.37 — 12.52)	0.695	2.06 (-7.7 — 11.82)	0.680
Age								
18-55 (reference)								
≥ 56	-8.41 (-17.05 — -0.24)	**0.050**	-7.3 (-22.94 — 8.34)	0.357	1.17 (-7.42 — 9.76)	0.788	1.03 (-6.99 — 9.05)	0.803
Single								
No (reference)								
Yes	-8.17 (-18.77 — 2.43)	0.130	-12.05 (-31.23 — 7.13)	0.216	-5.76 (-16.3 — 4.77)	0.281	-6.49 (-16.33 — 3.35)	0.198
What is your level of education?								
No education / Primary school graduate (reference)								
Middle school / High school graduate	-2.19 (-13.65 — 9.27)	0.706	-33.64 (-54.37 — -12.91)	**0.002**	0.98 (-10.41 — 12.36)	0.866	5.31 (-5.33 — 15.95)	0.329
University degree / Mater / PhD	-0.87 (-14.01 — 12.26)	0.896	-13.63 (-37.4 — 10.13)	0.259	1.69 (-11.35 — 14.74)	0.798	4.27 (-7.92 — 16.46)	0.493
How long have you been on hemodialysis?								
≤ 6 months (reference)								
6 months - 5 years	17.35 (0.8 — 33.9)	0.088	11.14 (-18.8 — 41.07)	0.463	23.43 (6.99 — 39.87)	**0.006**	7.23 (-8.14 — 22.6)	0.358
≥ 5 years	15.44 (-1.39 — 32.28)	0.072	6.82 (-23.63 — 37.28)	0.658	15.79 (-0.94 — 32.51)	0.064	-2.49 (-18.11 — 13.13)	0.755
What type of vascular access do you currently have?								
Arteriovenous fistula (reference)								
Graft	-10.51 (-31.24 — 10.22)	0.318	-9.21 (-46.71 — 28.29)	0.628	-2.22 (-22.81 — 18.37)	0.832	-5.85 (-25.1 — 13.4)	0.552
Temporary dialysis catheter	-4.13 (-25.32 — 17.07)	0.701	4.44 (-33.91 — 42.79)	0.819	8.86 (-12.2 — 29.92)	0.407	-9.22 (-28.9 — 10.46)	0.360
Permanent dialysis catheter	-12.04 (-25.91 — 1.83)	**0.040**	8.22 (-16.87 — 33.32)	0.518	-2.19 (-15.97 — 11.59)	0.754	-7.89 (-20.77 — 4.99)	0.232
Are you satisfied with your vascular access?								
Very (reference)								
Quite	-8.26 (-16.88 — 0.36)	0.060	-11.63 (-27.21 — 3.96)	0.143	-7.29 (-15.85 — 1.27)	0.095	-11.02 (-19.02 — -3.02)	**0.008**
A little / Not at all	-6.77 (-25.72 — 12.19)	0.481	-24.86 (-59.15 — 9.42)	0.154	-27.09 (-45.92 — -8.26)	**0.005**	-17.3 (-34.9 — -0.3)	**0.046**
Which vascular access is considered appropriate for you?								
Arteriovenous fistula (reference)								
Graft	-1.56 (-21.1 — 17.97)	0.874	7.8 (-27.55 — 43.15)	0.663	-7.89 (-27.3 — 11.52)	0.423	0.33 (-17.8 — 18.46)	0.972
Temporary dialysis catheter	2.17 (-27.98 — 32.31)	0.887	22.11 (-32.43 — 76.64)	0.424	7.6 (-22.35 — 37.55)	0.616	16.36 (-11.63 — 44.35)	0.254
Permanent dialysis catheter	-6.86 (-21.91 — 8.2)	0.369	-17.24 (-44.48 — 10)	0.213	-1.56 (-16.51 — 13.4)	0.837	2.32 (-11.65 — 16.29)	0.745
Adjusted R^2	0.12		0.06		0.09		0.09	
Durbin-Watson d	2.01		1.93		2.12		2.21	

β:regression coefficient 95% CI: 95% Confidence Interval

Table S3. Results of multiple linear regression analysis with dependent variables of the dimensions of SF-36 scale (continue).

	Vitality		Social function		Role emotional		Mental health	
	β (95% CI)	P	β (95% CI)	P	β (95% CI)	P	β (95% CI)	P
Gender								
Male (reference)								
Female	-1.88 (-7.73 — 3.96)	0.525	-0.15 (-7.8 — 7.5)	0.969	-0.84 (-16.26 — 14.58)	0.914	-2.72 (-8.92 — 3.47)	0.386
BMI								
Normal (reference)								
Overweight	2.57 (-3.79 — 8.92)	0.426	0.12 (-8.21 — 8.45)	0.977	1.3 (-15.48 — 18.07)	0.879	2.32 (-4.42 — 9.06)	0.497
Obese	0.47 (-6.86 — 7.81)	0.898	-7.98 (-17.58 — 1.62)	0.102	-13.02 (-32.36 — 6.33)	0.185	1.06 (-6.71 — 8.83)	0.788
Age								
18-55 (reference)								
≥ 56	-0.74 (-6.77 — 5.29)	0.809	-3.41 (-11.3 — 4.49)	0.395	-4.5 (-20.41 — 11.41)	0.577	1.83 (-4.56 — 8.22)	0.572
Sinlge								
No (reference)								
Yes	-6.12 (-13.52 — 1.27)	0.104	-6.57 (-16.26 — 3.11)	0.182	-13.87 (-33.39 — 5.65)	0.162	-4.5 (-12.34 — 3.34)	0.258
What is your level of education?								
No education / Primary school graduate (reference)								
Middle school / High school graduate	3.35 (-4.64 — 11.34)	0.409	0.01 (-10.46 — 10.47)	0.999	-23.96 (-45.05 — -2.86)	**0.026**	5.91 (-2.57 — 14.38)	0.170
University degree / Mater / PhD	3.49 (-5.67 — 12.65)	0.452	7.03 (-4.97 — 19.02)	0.249	-6.44 (-30.61 — 17.74)	0.599	10.3 (0.58 — 20.01)	**0.038**
How long have you been on hemodialysis?								
≤ 6 months (reference)								
6 months - 5 years	4.5 (-7.04 — 16.04)	0.442	12.66 (-2.45 — 27.78)	0.100	-2.57 (-33.03 — 27.89)	0.868	-1.21 (-13.45 — 11.02)	0.845
≥ 5 years	0.52 (-11.22 — 12.26)	0.931	7.56 (-7.82 — 22.94)	0.333	-0.45 (-31.44 — 30.54)	0.977	-3.64 (-16.09 — 8.81)	0.564
What type of vascular access do you currently have?								
Arteriovenous fistula (reference)								
Graft	-11.65 (-26.11 — -2.81)	**0.035**	-6.14 (-25.07 — 12.8)	0.523	5.23 (-32.92 — 43.38)	0.787	-1.88 (-17.21 — 13.44)	0.808
Temporary dialysis catheter	-1.42 (-16.21 — 13.36)	0.849	-2.65 (-22.01 — 16.72)	0.787	22.67 (-16.34 — 61.68)	0.252	-2.38 (-18.06 — 13.29)	0.764
Permanent dialysis catheter	-4.67 (-14.35 — 5)	0.341	-12.82 (-25.49 — -0.15)	**0.047**	7.95 (-17.58 — 33.48)	0.539	-2.99 (-13.25 — 7.26)	0.565
Are you satisfied with your vascular access?								
Very (reference)								
Quite	-9.99 (-16 — -3.98)	**0.001**	-12.91 (-20.78 — -5.04)	**0.001**	-7.54 (-23.4 — 8.32)	0.349	-6.29 (-12.67 — -0.08)	**0.050**
A little / Not at all	-7.26 (-20.48 — 5.95)	0.279	-9.94 (-27.25 — 7.37)	0.258	-24.47 (-59.35 — 10.42)	0.168	-12.34 (-26.35 — 1.67)	0.084
Which vascular access is considered appropriate for you?								
Arteriovenous fistula (reference)								
Graft	4.96 (-8.66 — 18.59)	0.472	-6.4 (-24.24 — 11.45)	0.480	-5.65 (-41.61 — 30.31)	0.756	-2.76 (-17.21 — 11.68)	0.706
Temporary dialysis catheter	8.01 (-13.01 — 29.03)	0.452	13.22 (-14.31 — 40.75)	0.344	37.44 (-18.04 — 92.92)	0.184	3.92 (-18.37 — 26.21)	0.729

Permanent dialysis catheter	1.11 (-9.38 — 11.61)	0.834	-0.78 (-14.53 — 12.97)	0.911	-21.56 (-49.27 — 6.15)	0.126	-1.13 (-12.27 — 10)	0.841
Adjusted R^2	0.08		0.16		0.04		0.04	
Durbin-Watson d	1.98		1.92		1.95		2.06	

β:regression coefficient 95% CI: 95% Confidence Interval

Table S3. Results of multiple linear regression analysis with dependent variables of the dimensions of SF-36 scale (continue).

	PCS		MCS	
	β (95% CI)	P	β (95% CI)	P
Gender				
Male (reference)				
Female	-1.18 (-4.36 – 2.00)	0.468	-0.53 (-3.78 – 2.72)	0.752
BMI				
Normal (reference)				
Overweight	-0.65 (-4.1 – 2.8)	0.715	1.53 (-2.02 – 5.08)	0.399
Obese	-0.64 (-4.62 – 3.34)	0.754	-1.55 (-5.65 – 2.55)	0.458
Age				
18-55 (reference)				
≥ 56	-1.99 (-5.26 – 1.28)	0.235	0.46 (-2.91 – 3.83)	0.787
Sinlge				
No (reference)				
Yes	-3.05 (-7.07 – 0.97)	0.139	-2.97 (-7.09 – 1.15)	0.161
What is your level of education?				
No education / Primary school graduate (reference)				
Middle school / High school graduate	-2.4 (-6.73 – 1.93)	0.281	0.19 (-4.26 – 4.64)	0.934
University degree / Mater / PhD	-1.68 (-6.66 – 3.3)	0.508	3.65 (-1.47 – 8.77)	0.164
How long have you been on hemodialysis?				
≤ 6 months (reference)				
6 months - 5 years	8.74 (2.47 – 15.01)	**0.007**	-1.81 (-8.24 – 4.62)	0.583
≥ 5 years	5.84 (-0.53 – 12.21)	0.075	-2.51 (-9.06 – 4.04)	0.454
What type of vascular access do you currently have?				
Arteriovenous fistula (reference)				
Graft	-4.14 (-12 – 3.72)	0.303	-0.34 (-8.4 – 7.72)	0.934
Temporary dialysis catheter	-1.29 (-9.33 – 6.75)	0.754	1.82 (-6.43 – 10.07)	0.666
Permanent dialysis catheter	-2.76 (-8.01 – 2.49)	0.304	-0.77 (-6.16 – 4.62)	0.781
Are you satisfied with your vascular access?				
Very (reference)				
Quite	-3.97 (-7.24 – -0.7)	**0.019**	-3.73 (-7.08 – -0.38)	**0.031**
A little / Not at all	-6.76 (-13.93 – 0.41)	0.067	-5.72 (-13.09 – 1.65)	0.131
Which vascular access is considered appropriate for you?				
Arteriovenous fistula (reference)				
Graft	0.26 (-7.15 – 7.67)	0.946	-1.5 (-9.1 – 6.1)	0.699
Temporary dialysis catheter	3.16 (-8.27 – 14.59)	0.589	7 (-4.72 – 18.72)	0.244
Permanent dialysis catheter	-1.57 (-7.27 – 4.13)	0.589	-1.74 (-7.6 – 4.12)	0.560
Adjusted R^2	0.09		0.02	
Durbin-Watson d	2.01		1.90	

β:regression coefficient 95% CI: 95% Confidence Interval

Table S4. Results of multiple linear regression analysis with dependent variables of the dimensions of MVQoL scale

	Symptoms		Function		Interpersonal relationships	
	β (95% CI)	P	β (95% CI)	P	β (95% CI)	P
Gender						
Male (reference)						
Female	-2.56 (-5.12 — 0)	0.050	-1.7 (-4.59 — 1.19)	0.246	1.49 (-1.85 — 4.83)	0.380
BMI						
Normal (reference)						
Overweight	0.11 (-2.67 — 2.9)	0.937	0.1 (-3.05 — 3.25)	0.950	-0.01 (-3.65 — 3.62)	0.995
Obese	-1.2 (-4.41 — 2.01)	0.460	-4.97 (-8.6 — -1.35)	**0.008**	-1.83 (-6.02 — 2.37)	0.391
Age						
18-55 (reference)						
≥ 56	0.94 (-1.7 — 3.58)	0.484	1.01 (-1.98 — 3.99)	0.506	1.79 (-1.66 — 5.23)	0.307
Sinlge						
No (reference)						
Yes	-1.5 (-4.74 — 1.74)	0.362	0.02 (-3.64 — 3.68)	0.992	-10.17 (-14.4 — -5.94)	<0.001
What is your level of education?						
No education / Primary school graduate (reference)						
Middle school / High school graduate	6.45 (2.95 — 9.95)	<0.001	-1.12 (-5.08 — 2.83)	0.575	3.31 (-1.26 — 7.88)	0.154
University degree / Mater / PhD	6.64 (2.63 — 10.66)	0.001	2.28 (-2.25 — 6.82)	0.321	1.41 (-3.83 — 6.64)	0.596
How long have you been on hemodialysis?						
≤ 6 months (reference)						
6 months - 5 years	-1.36 (-6.41 — 3.7)	0.597	0.45 (-5.26 — 6.16)	0.877	2.61 (-3.99 — 9.21)	0.435
≥ 5 years	-2.03 (-7.18 — 3.11)	0.436	2.69 (-3.12 — 8.5)	0.361	2.69 (-4.03 — 9.4)	0.430
What type of vascular access do you currently have?						
Arteriovenous fistula (reference)						
Graft	0.2 (-6.14 — 6.53)	0.951	-2.01 (-9.16 — 5.15)	0.580	0.33 (-7.94 — 8.59)	0.938
Temporary dialysis catheter	-3.38 (-9.86 — 3.1)	0.304	4.79 (-2.53 — 12.1)	0.198	-1.13 (-9.59 — 7.32)	0.792
Permanent dialysis catheter	-1.58 (-5.82 — 2.66)	0.461	-1.72 (-6.51 — 3.07)	0.478	-1.92 (-7.46 — 3.61)	0.493
Are you satisfied with your vascular access?						
Very (reference)						
Quite	-2.34 (-4.97 — 0.29)	0.081	1.61 (-1.36 — 4.59)	0.285	-2.58 (-6.01 — 0.86)	0.140
A little / Not at all	-2.47 (-8.27 — 3.32)	0.400	5.16 (-1.38 — 11.7)	0.121	-8.01 (-15.57 — -0.45)	0.038
Which vascular access is considered appropriate for you?						
Arteriovenous fistula (reference)						
Graft	-1.13 (-7.1 — 4.84)	0.708	-1.2 (-7.94 — 5.54)	0.726	-2.38 (-10.17 — 5.42)	0.548
Temporary dialysis catheter	-2.97 (-12.18 — 6.24)	0.525	2.08 (-8.32 — 12.49)	0.693	1.54 (-10.49 — 13.56)	0.801
Permanent dialysis catheter	3.1 (-1.5 — 7.7)	0.185	0.64 (-4.56 — 5.83)	0.809	2.13 (-3.88 — 8.13)	0.485
Adjusted R^2	0.14		0.06		0.14	
Durbin-Watson d	2.09		2.02		1.98	

β:regression coefficient 95% CI: 95% Confidence Interval

Table S4. Results of multiple linear regression analysis with dependent variables of the dimensions of MVQoL scale (continue)

	Emotional well-being		Transcendence		Overall quality of life score	
	β (95% CI)	P	β (95% CI)	P	β (95% CI)	P
Gender						
Male (reference)						
Female	3.14 (-1.86 – 8.14)	0.217	-2.81 (-6.86 – 1.24)	0.172	-0.25 (-1.26 – 0.77)	0.634
BMI						
Normal (reference)						
Overweight	4.12 (-1.32 – 9.55)	0.137	0.3 (-4.11 – 4.7)	0.894	0.46 (-0.65 – 1.57)	0.411
Obese	2.28 (-3.98 – 8.54)	0.472	-1.16 (-6.23 – 3.91)	0.651	-0.69 (-1.96 – 0.59)	0.287
Age						
18-55 (reference)						
≥ 56	3.33 (-1.81 – 8.47)	0.202	-1.75 (-5.91 – 2.41)	0.406	0.53 (-0.52 – 1.58)	0.318
Sinlge						
No (reference)						
Yes	4 (-2.3 – 10.31)	0.211	-6.08 (-11.19 – -0.98)	**0.020**	-1.37 (-2.66 – -0.09)	**0.036**
What is your level of education?						
No education / Primary school graduate (reference)						
Middle school / High school graduate	1.67 (-5.16 – 8.5)	0.629	0.55 (-4.98 – 6.08)	0.843	1.09 (-0.3 – 2.48)	0.125
University degree / Mater / PhD	4.78 (-3.03 – 12.59)	0.228	1.74 (-4.59 – 8.06)	0.588	1.68 (0.09 – 3.27)	**0.038**
How long have you been on hemodialysis?						
≤ 6 months (reference)						
6 months - 5 years	6.2 (-3.65 – 16.04)	0.215	0.15 (-7.82 – 8.12)	0.970	0.8 (-1.2 – 2.81)	0.429
≥ 5 years	2.62 (-7.38 – 12.63)	0.605	-2.24 (-10.35 – 5.86)	0.585	0.37 (-1.66 – 2.41)	0.718
What type of vascular access do you currently have?						
Arteriovenous fistula (reference)						
Graft	-3.65 (-15.97 – 8.68)	0.559	-0.24 (-10.21 – 9.74)	0.963	-0.54 (-3.04 – 1.97)	0.673
Temporary dialysis catheter	-5.52 (-18.13 – 7.09)	0.388	5.45 (-4.76 – 15.67)	0.293	0.02 (-2.55 – 2.59)	0.987
Permanent dialysis catheter	-3.06 (-11.31 – 5.18)	0.464	2.93 (-3.75 – 9.6)	0.388	-0.54 (-2.22 – 1.14)	0.528
Are you satisfied with your vascular access?						
Very (reference)						
Quite	-4.73 (-9.86 – 0.41)	0.071	-6.4 (-10.56 – -2.25)	**0.003**	-1.44 (-2.49 – -0.4)	**0.007**
A little / Not at all	-9.76 (-21.03 – 1.51)	0.089	-15.84 (-24.96 – -6.72)	**0.001**	-3.09 (-5.39 – -0.8)	**0.009**
Which vascular access is considered appropriate for you?						
Arteriovenous fistula (reference)						
Graft	8.32 (-3.29 – 19.93)	0.159	-4 (-13.41 – 5.4)	0.401	-0.04 (-2.4 – 2.33)	0.974
Temporary dialysis catheter	7.61 (-10.31 – 25.54)	0.402	1.05 (-13.46 – 15.56)	0.887	0.93 (-2.72 – 4.58)	0.615
Permanent dialysis catheter	1.76 (-7.24 – 10.77)	0.699	-8.59 (-15.88 – -1.3)	**0.021**	-0.1 (-1.93 – 1.74)	0.916
Adjusted R^2	0.02		0.15		0.11	
Durbin-Watson d	1.86		1.73		1.7	

β:regression coefficient 95% CI: 95% Confidence Interval
